# ‘It’s all settled on the right page’ surrogates’ feelings and reflections of surrogacy two decades on

**DOI:** 10.1093/humrep/deae216

**Published:** 2024-09-20

**Authors:** K Shaw, S Imrie, P Hall, V Jadva

**Affiliations:** Centre for Family Research, University of Cambridge, Cambridge, UK; Centre for Family Research, University of Cambridge, Cambridge, UK; Murray Edwards College, University of Cambridge, Cambridge, UK; Centre for Family Research, University of Cambridge, Cambridge, UK; Centre for Family Research, University of Cambridge, Cambridge, UK; Department of Psychology, City, University of London, London, UK

**Keywords:** surrogacy, qualitative, intended parents, surrogates, traditional surrogacy, gestational surrogacy, genetic, relationships

## Abstract

**STUDY QUESTION:**

How do surrogates think, feel, and reflect on their experiences of being a surrogate over time?

**SUMMARY ANSWER:**

Despite continuing to require physical, emotional, and interpersonal labour, surrogates in this study reflected positively on their experiences of being a surrogate decades later.

**WHAT IS KNOWN ALREADY:**

Research on families created through surrogacy shows that it can be a positive experience for both the intended parents and the surrogate. No existing research has examined the experiences of surrogacy for surrogates beyond 10 years post-birth.

**STUDY DESIGN, SIZE, DURATION:**

Semi-structured, qualitative interviews were conducted with 21 women who had completed a total of 71 surrogacy arrangements. Participants had given birth to their first surrogate child on average 20 years previously.

**PARTICIPANTS/MATERIALS, SETTINGS METHODS:**

Data were collected about (i) surrogates’ relationships with the families they had helped, (ii) how important being a surrogate was to their own identity, and (iii) how they felt surrogacy was perceived by the public. Data were analysed according to the principles of reflexive thematic analysis.

**MAIN RESULTS AND THE ROLE OF CHANCE:**

Surrogates’ reflections on their experiences were constructed into two themes: (i) hard work but worth it and (ii) part of who I am. Surrogacy had taken a physical toll on some participants, and for most, being a surrogate continued to involve emotional labour and effort to maintain relationships with the families. Making friends through the process and feeling proud of what they had done to help someone meant that overall, surrogates reflected positively on surrogacy and had incorporated their experiences as surrogates into a stable aspect of their identity.

**LIMITATIONS, REASONS FOR CAUTION:**

There is a risk of selection bias, with surrogates who had a more positive experience being more likely to continue to participate in the research. All surrogacies occurred within the UK. While the sample is relatively diverse in terms of surrogates’ ages, current employment status, and relationship status, the sample lacks ethnic diversity. Most participants had been surrogates for heterosexual couples, and thus long-term relationships involving gay couples or single men may differ.

**WIDER IMPLICATIONS OF THE FINDINGS:**

The findings from this study show the importance of understanding surrogacy as a ‘relational’ process and suggest to prospective surrogates and intended parents what they may expect from their relationship over time. Given the findings that even as the children grow up, being a surrogate continues to require emotional labour, support should be made available to surrogates over the longer term if required.

**STUDY FUNDING/COMPETING INTEREST(S):**

This project was funded by the Wellcome Trust [grant number 208013/Z/17/Z] and the University of Cambridge Returning Carers Scheme. The authors have no competing interests to declare.

**TRIAL REGISTRATION NUMBER:**

N/A.

## Introduction

Whilst recent years have seen an increase in the practice of surrogacy globally, very little is understood about the longer-term experiences of women who gestate pregnancies for someone else. Regulating surrogacy is ethically complex because there is the risk of surrogates being exploited, the possibility of physical or psychological problems for the surrogate, parents, and/or child, and because reproduction involving a third-party challenges traditional, two-parent family ideals (Nuffield Council on Bioethics, 2023). As such, it is currently illegal for organizations in the UK to take payment for assisting intended parents and surrogates through the process, and the surrogate retains parental rights over the child from birth until the parental order is granted in court (usually 6+ months following the birth).

Undergoing surrogacy can be a positive experience for both the intended parents (Horsey *et al.*, 2023) and the surrogate (Imrie and Jadva, 2014). Additionally, children born through surrogacy are found to be well-adjusted and functioning well (Söderström-Anttila *et al.*, 2016; Golombok, 2020). Findings relating to surrogates’ psychological health are also encouraging, with surrogates in the UK and USA reporting relatively low rates of depression in the weeks following the birth (Parkinson *et al.*, 1998; Söderström-Anttila *et al.*, 2016), and at 6 months (van den Akker, 2007), 1 year (Jadva *et al.*, 2003), and 10 years postpartum (Imrie and Jadva, 2014). Finally, studies of surrogates from a range of different countries have shown that surrogates do not see the child they carry as their own (Jadva *et al.*, 2003; Teman, 2010; Hibino and Shimazono, 2013; Lamba *et al.*, 2018; Kneebone *et al.*, 2022). From a physical health perspective, a recent systematic review of surrogacy in commercial contexts reported that surrogates do experience physical challenges, such as pain, pregnancy complications, and negative side effects of hormone injections during treatment (Kneebone *et al.*, 2022). To our knowledge, no study has obtained information from surrogates about their longer-term (beyond 10 years) reflections on how being a surrogate has impacted their health and life experiences.

Existing research and legal guidance for the regulation of surrogacy has traditionally focused on assessments of physical and psychological health of the intended parents and the surrogates in isolation from each other (Gunnarsson Payne *et al.*, 2020). However, there has been a shift towards recognizing that surrogacy happens within cultural contexts that are shaped by social ideals of parenthood and kinship (Shah *et al.*, 2022). Following a systematic review of the qualitative literature, Gunnarsson Payne *et al.* (2020) called for surrogacy organizations and regulatory bodies to acknowledge that the relationships between surrogates, intended parents, and the child shape the experience of surrogacy for each party, describing surrogacy as a ‘relational process’. Two reviews of the surrogacy literature have reported that, for both international and domestic surrogacy arrangements, maintaining positive relationships between the surrogate and intended parents is consistently found to be beneficial for both parties (Gunnarsson Payne *et al.*, 2020; Kneebone *et al.*, 2022). Yee *et al.*, (2023) have suggested that these relationships can also be protective against difficulties that arise during the surrogacy journey, for example, positive support from intended parents helped surrogates who experienced mental health challenges during the coronavirus (COVID-19) pandemic. The opportunity to have a meaningful relationship with their surrogate was a key motivator for intended parents who elected to undergo surrogacy in the UK (Jadva *et al.*, 2021). Both surrogates (Imrie and Jadva, 2014) and intended parents (Horsey *et al.*, 2023) in the UK report that they remained in contact with each other as the child(ren) reach middle-childhood.

Considering these findings, there has been a call for changes to the regulation of surrogacy in the UK to make the domestic process more accessible for all parties (Jackson, 2016). Following a public consultation, the Law Commission of England and Wales and the Scottish Law Commission published their recommendations for surrogacy law reform in March 2023, including the proposal that the intended parents are the registered parents of the child from birth, thus removing one of the key motivators for intended parents travelling abroad to undergo surrogacy (Jadva *et al.*, 2019). Surrogates are found to be generally in favour of these proposed changes (Horsey *et al.*, 2022).

Despite the increasing numbers of children born through surrogacy in the UK and internationally, we still understand very little about the longer-term experiences of surrogacy for the surrogates themselves. Does their relationship with the parents change over time? Do they continue to hold positive views of surrogacy? In 2003, findings were reported from 34 surrogates who had given birth to a child through surrogacy 1 year previously (Jadva *et al.*, 2003). The women were then revisited 10 years following the birth, with additional women recruited to the study to maintain the sample size (Imrie and Jadva, 2014). The original study found that surrogates were in contact with 79% of couples and 76% of children a year after the birth. Surrogates reported forming strong relationships with some of the couples they helped, although frequency of contact reduced over time. At the 10-year study, surrogates had remained in contact with 85% of mothers and 77% of children, and their contact and quality of relationships with the family did not differ according to the type of surrogacy undertaken (Imrie and Jadva, 2014). The second phase of the study also found that 24 of the 34 surrogates had undergone repeated surrogacy pregnancies, with a mean of 3 pregnancies (range 1–8). The current study revisits surrogates 10 years after the second phase of the project (i.e. ∼20 years after the birth of the child) to understand how surrogates think, feel, and reflect on their experiences of being a surrogate up to two decades later.

## Materials and methods

### Participants

This article reports on interviews with 21 surrogates in the UK who had completed a total of 71 surrogacy arrangements (Median = 3; range 1–6; *M *=* *3.38; SD* *=* *1.8, see Table 1). Participants had taken part in a previous study and/or follow-up study on surrogates’ psychological health and experiences (Imrie and Jadva, 2014; Jadva *et al.*, 2015). The initial sample seen at Phase 1 was recruited via an ongoing longitudinal study and a UK surrogacy organization (Jadva *et al.*, 2003). In the second phase of the study, additional surrogates (N = 14) were recruited through surrogacy organizations and fertility clinics (Imrie and Jadva, 2014). Contact was attempted with all 34 surrogates from the second phase of the study (Imrie and Jadva, 2014) by phone and email. Ten participants were uncontactable, one participant arranged an interview but did not attend, and two participants declined to take part due to time constraints (88% retention rate for contactable participants from Phase 2 of the study).

**Table 1.** Sociodemographic information.

	N	%
Relationship status		
Married	10	48
Cohabiting with partner	5	24
Single	6	28
Surrogate working status		
Full-time	11	52
Not currently working	5	24
Part-time	2	10
Retired	2	10
Part-time student, part-time work	1	5
Occupation		
Managerial/technical	2	10
Skilled non-manual	4	19
Skilled manual	3	14
Partly skilled	7	33
N/A not in work	5	24
No. of surrogate births		
1	5	24
2	2	10
3	4	19
4	3	14
5	4	19
6	3	14

Participants had undertaken their first (successful) surrogacy arrangement an average of 20 years ago (Mean 20.3 years, SD = 3.31, range 13–26 years) for a heterosexual couple. The sample included surrogates who had done (i) gestational or ‘host’ surrogacy arrangements (n = 10, 48%), where the surrogate did not use her egg for the pregnancy, (ii) genetic or ‘traditional’ surrogacy arrangements (n = 5, 24%), where the surrogate’s egg was used for the pregnancy, and (iii) both traditional and gestational surrogacy arrangements (n = 6, 29%). Two (10%) participants had been a surrogate for a family member, 1 (5%) had met the couple they helped via a friend, and the remaining 18 (85%) surrogates had organized at least one of their arrangements via a surrogacy organization. All arrangements that had been conducted prior to the second phase of the study involved either the intending parents’ gametes or the surrogate’s egg. Five surrogates had conducted surrogacy arrangements since Phase 2 of the study. Seven of these 11 pregnancies were gestational surrogacy arrangements; however, whether the intending parents’ egg or donor eggs were used was not recorded.

Surrogates’ mean age at the time of interviews was 52 years (SD = 7.30, range 40–73 years). All surrogates had their own children before they had undertaken their first surrogacy arrangement. Twenty (95%) participants identified as white. One (5%) participant identified as black African/Caribbean. Fourteen (66%) participants worked full or part-time. Most (N = 14, 67%) participants were married to or cohabiting with a partner (see Table 1).

### Measures

Semi-structured interviews were conducted with surrogates via video or phone call between December 2021 and September 2022. Interviews were conducted by one of two researchers (K.S. and P.H.) trained in administering the semi-structured interview used in the present study. The interviews lasted between 45 min and 2 h. Interviews were audio recorded, transcribed verbatim, and anonymized. Transcripts were checked for accuracy against the original audio recording during the data familiarization phase of analysis. Quotations in the results section are presented verbatim, although pseudonyms have been used to protect participant identity, ellipsis have been used when words have been omitted, and repeated words and pause fillers (‘er’, ‘um’) have been removed to aid readability. Following discussion and consensus from the authors, minor alterations have been made to some data to maintain participant anonymity (Saunders *et al.*, 2015).

The interview included sections asking participants about their physical and psychological health since the previous phase, their contact levels and relationships with the families they had helped, the extent to which they felt that surrogacy was important to their own identity, and how they felt surrogacy was perceived by the UK public. The content of the interview was influenced by previous phases of the study and by other relevant qualitative research exploring the experiences of families formed through assisted reproductive technologies (Imrie *et al.*, 2020). The current article focuses on surrogates’ feelings about, and reflections on, their surrogacy experiences.

### Analysis

Data were analysed by the first author, following the principles of reflexive thematic analysis (Braun and Clarke, 2022), which aims to understand patterns of meaning across a data set. A data-driven, inductive approach was taken to analysis because of the limited existing qualitative literature relating to the research question. This means the resulting themes are strongly linked to the data themselves rather than pre-existing coding schemes. Two sections of each interview (surrogates’ health and thoughts and feelings about surrogacy over time) were coded line-by-line using ATLAS.ti. Codes were arranged into initial groups based on the challenges and rewards experienced by surrogates at different time points on the surrogacy journey. Two candidate themes were constructed that related to (i) the work involved in surrogacy and (ii) the impact of being a surrogate on participants’ identity. Through discussion with the other authors and reengagement with the data, key themes and subthemes were finalized to ensure the analysis was reflective of the full dataset.

Through journaling and discussion between authors, we aimed to reflect on how our own position and experiences shape data collection and analysis (Braun and Clarke, 2019). None of the authors have been surrogates, so may be seen by participants as ‘outsiders’ (Hayfield and Huxley, 2015). The authors recognize that their existing position as people who are supportive of inclusive family-building will have shaped the study design and analysis (Braun and Clarke, 2019). In response, Gaskell and Bauer’s (2000) criteria for confidence and relevance were adhered to at the analysis phase to ensure the reported results reflect the reality of the data and offer relevant information to the people involved, namely current and prospective surrogates and intended parents, policy makers, and other researchers.

Written, informed consent was collected from all participants, and ethical approval was obtained from the University of Cambridge Psychology Research Ethics Committee.

## Results

The results centre on two themes: (i) Hard work but worth it and (ii) part of who I am. Both themes answer the research question: how do surrogates think, feel, and reflect on their experience of being a surrogate over time?

### Theme 1: hard work but worth it

This theme describes the ongoing physical, interpersonal, and emotional challenges involved in being a surrogate, along with the positive experiences throughout the journey that make surrogates feel these difficulties are worth it. Put succinctly by Adrianna: ‘*people think it’s easy and it’s not… it’s bloody hard work*’.

#### Physical toll

Some participants had previously enjoyed straightforward pregnancies when forming their own families, so they felt able to help others through surrogacy:

*I sailed through my pregnancy and the birth for my daughter, and I thought ‘why wouldn’t I help another woman’*—Annette

Other participants experienced medical complications and sickness during pregnancy: ‘*pregnancy is not necessarily always straightforward, it is painful… there are risks*’ (Harriet). A few surrogates reported traumatic births during surrogacy that meant they did not attempt any further pregnancies (‘*I hated being pregnant… I nearly died*’—Rhea). Surrogates also described feeling more aware of changes to their appearance following a surrogate pregnancy compared to when they had their own children. This was because they did not have a baby with them to justify their change in appearance to others:

*I have destroyed my body. I look like a burst sofa but I can’t do anything about it… When you’ve had a baby and you’ve got your baby and you’re a significantly different shape, people excuse you for that change in shape. But when you haven’t got the baby… I definitely felt more self-conscious about my appearance. The more pregnancies I’ve had, the harder it has been to get back into shape*.—Rhea

Several participants were living with physical health problems that had been caused by undergoing multiple pregnancies. Despite this physical toll, they were still happy with their decision to have carried the surrogate pregnancies:

*I’ve done damage to my hips, probably through being pregnant so many times in ten years. It was hard on my body and I’m now suffering because of it. Whether that means I would have changed it, I probably wouldn’t, because at the time it was what I wanted to do, and it was great—*Annette

For many participants, the physical toll involved in surrogacy was felt to be worth it because they found it highly rewarding to bring new life into the world:

*I saw this little family together for the first time and they were both crying, and the baby was crying and the midwife was crying… everyone was crying, and I remember thinking in that exact moment “that’s why I did this”. That’s made the entire nine months of pregnancy and sickness worth it.*—Rose

#### Managing relationships

Just over three-quarters of surrogates who had maintained contact up until the previous phase of the study (children aged around 10 years) remained in contact at this phase. Managing these relationships over time had taken work from the surrogates:

*It’s definitely much more involved than just sort of getting pregnant, having a baby, see you later*.—Susie

For example, during the pregnancy, some described feeling frustrated with the intended parents because they were in contact too frequently. As Yvonne describes, managing the relationship with the intended parents during the pregnancy can be a challenge, but this is all part of the experience of being a surrogate:

*I felt they [the couple] were quite nervous about it which just irritated me… Towards the last few days before the birth they want to be close by and they’re so excited, and I’m just like ‘I can’t be bothered’. But then you have to be considerate and make those adjustments to suit other people—*Yvonne

In the years that had passed since the birth, participants described ups and downs in the relationships with the families they had helped. When Gail agreed to carry a sibling for a family she had already helped via a gestational arrangement, she anticipated that she would carry on seeing the family regularly, so she was happy to be a traditional surrogate the second time around. However, when the family moved away from the area, she found that she missed the second child who she shared a genetic relationship with. She described the complexity of this relationship, saying ‘*he is my child but he’s not my child sort of thing*’ (Gail). Conversely, Belinda experienced challenges in a relationship with a parent who was ‘*too pushy*’ and wanted more contact from her than she wanted to give. Some surrogates reported that the COVID-19-related lockdowns had impacted how much contact they were able to have with the families.

Surrogates also described having different relationships with the families from different arrangements. For example, Rhea said that ‘*I talk more with some [families] than others*’, and this depended on how much she had in common with them as people. Some surrogates would seek intended parents with whom they got along well:

*[The couple] were like-minded and I got along with them as friends prior to going on our journey*—Ruby.

Managing relationships with the families required ongoing work for surrogates, but participants felt that the friends they had made through the process was one of the most rewarding aspects of being a surrogate. In some cases, the relationship with the parents had become very close, or even familial, and the surrogates and their own families continued to benefit from these positive relationships:

*The best bits are building really amazing friendships with people that have enriched my kids’ lives… My intended parents are like family to my kids. I feel like I’ve built this network of people who absolutely adore my kids, and who my kids look up to. And for me that’s been really rewarding*.—Hazel

Gloria and Eloise, who carried pregnancies for family members, described how the relationships they have with the child bring them a great deal of joy:

*I’ve had a lot from it. I’ve had as much from being a surrogate as… I’ve got it tenfold. to be part of the family, watching them having a happy time and that. I just love it*.—Eloise

Later down the line, the work continued as surrogates were required to join in conversations with the child about their role in their conception:

*I did have a chat with him because his mum asked me to talk to him, and I showed him different magazines and all the articles and sort of, helped him understand what it was all about.*—Susie

Although the relationships between the surrogate, parents, and the child/young adult had often involved ‘*a few upsets here and there*’ (Clara), most surrogates reflected that at this time point, they were confident the relationship would be sustained:

*If the relationship had broken down early on, that would have been quite difficult. But by now, this far down the line I don’t think it’s going to, I can’t see a reason we can’t stay in contact forever. It’s very satisfying to know it’s all settled on the right page.*—Emilia

#### Emotional labour

Surrogates reflected on becoming and being a surrogate as an emotional experience, and for many, the emotional work continued even two decades later. For example, the impact of hormonal changes on their emotions at the time of the birth(s) remained a powerful memory for Hannah, who recalled feelings of emptiness following the birth, because her body had become ready to breastfeed and care for a baby, and her mind could not override this bodily sensation:

*It’s a feeling of emptiness… like ‘where did this baby go?’ Naturally as a mother, your body pines for that baby. Your boobs do, and everything else does. All your hormones are all over the place and not having the baby there kind of messes everything up. It’s not that you want the child or anything like that… It’s your body, not your mind, pining for the child.*—Hannah

Several surrogates remembered handing over the baby as an emotionally difficult process because of their hormones following the birth, but this emotional reaction was not conflated with thoughts of wanting to keep the baby:

*I was always really strong about it, but obviously the hormones did kick in and I did get upset for a few days afterwards. But I just remembered it’s something I really wanted to do. It didn’t take long to accept it. I think having the contact with [the couple] probably helped as well… obviously I did get upset but at no point did I want to keep [the baby]—*Zoe

Zoe reflected that maintaining contact with the family had helped manage her emotions, but other participants elected to take space from the family to help them emotionally distance themselves from the child:

*I distanced myself from him, and from babies and that. I just needed like a period of time of not seeing him and not going there—*Eloise

However, the majority of surrogates reflected on the handover as ‘*a joyous, happy moment*’ (Belinda) that was highly rewarding for them:

*I think the best way I’ve ever described that moment to anyone is that it’s like when you give someone a really cool present that you can’t wait to see them open… You get as much out of giving it as they get out of receiving it. It was like that.*—Rose

A few months after the birth, surrogates had to assist the intended parents to obtain the parental order, and this was remembered as being stressful for some:

*When we went to court, they [the judge] said “…if we decide to give you [the intended parents] this parental order…” and I thought gosh… if? If? I didn’t even know that there would even be an ‘if’! It’s quite scary—*Harriet

As the children grew up, some surrogates described continuing to feel some emotional responsibility for the child, because without their involvement, the child ‘*wouldn’t have been brought into this world*’ (Hazel). Even among the quarter who were no longer in contact with the child continued to wonder about their wellbeing. As Sheila put it, ‘*how are they and what are they up to today?*’. One surrogate expressed feeling guilty in an instance where the couple had separated since the child was born, despite recognizing that the negative impact it was having on the child was not to do with her, or the use of surrogacy:

*So many couples now don’t stay together. Just because they had a baby in a different way doesn’t make them different does it… But [child] has been affected a lot by the split, and I find that difficult—*Susie

Finally, some participants described continuing to manage worries about encountering negative reactions from others due to being a surrogate: ‘*I thought people would be talking behind my back thinking I’m a bad parent… who gives her babies away*’ (Lilian). Although Lilian’s worries were not realized, some surrogates reported continuing to experience stigma when they talk about surrogacy two decades later, and this was emotionally difficult for them: ‘*answering a barrage of personal questions… is actually quite tiring*’ (Hazel). Belinda also described a recent experience of discrimination in a job interview when she bought up her experience of surrogacy: ‘*[the interviewer] told me she would have put [my application] in the bin… she said “surrogacy doesn’t sit and feel right with me” and it became a big debate… I got up and walked out.*’ (Belinda).

Because of the physical, emotional, and interpersonal challenges managed by surrogates during the surrogacy journey, many described feeling ‘*proud of what I’ve done*’ (Belinda). For Ruby, aside from having her own children, she felt surrogacy had been ‘*my life’s biggest achievement*’.

### Theme 2: part of who I am

This theme describes (i) the ways in which becoming a surrogate fitted into participants’ existing identity as the type of person who likes to help others, (ii) the mismatch between stereotypes surrounding surrogacy and the surrogates’ lived experiences, (iii) how surrogacy had changed them as a person, and (iv) how being a surrogate remained part of their identity.

#### Being ‘that sort of person’

Most participants identified themselves as having always been a kind person who tries to help others. Becoming a surrogate was understood as an act of kindness undertaken to help people in need:

*I’ve always been a very caring, kind, thoughtful person, and I think anybody that knows me isn’t surprised I did surrogacy because that’s the sort of person I am… I just want to wave a magic wand and make all the world okay.*—Harriet

Like Harriet, Rhea also described her friend being unsurprised when she told them she was going to be a surrogate, replying ‘*that’s so you*’ (Rhea). The participants acknowledged that they were in the minority in feeling that they could be a surrogate given that a common reaction from other women was that surrogacy was something they could not do themselves:

*It’s something that worked for me… I appreciate maybe the majority couldn’t do it and I get it, I understand it, that’s fine. But it worked for me*.—Belinda

For those who were members of a surrogacy organization, most participants knew other surrogates, and they described how all the surrogates they knew shared a passion for helping others:

*You’ve got to be that way inclined… I find that everybody I speak to has the same outlook about it as me. I see the same passion in them too*.—Adriana

Another trait the surrogates reported that they shared was a firm belief that when the time came, they would hand the baby over to the intended parents. This was a mindset that surrogates recognized someone ‘*either has or you don’t*’ (Eloise). Keeping the baby was never seen as an option by the participants: ‘*it’s something I could never even think about doing*’ (Belinda). Surrogates recognized that it must have been difficult for first-time intended parents to trust the surrogates’ conviction that they will hand over the baby:

*It must have been awful for [the intended parents]… putting all your trust in someone to have your child and give it back. On my side, I knew from the start that I would, and although they knew me, they couldn’t really know that*.—Marthe

Given that surrogates identified as people who wanted to help others, when age or health meant they could not carry another surrogate pregnancy, some participants found it difficult to have to stop. Instead, many found other ways to continue supporting new families and express their identity as a helpful person. For example, several participants had been involved in a surrogacy organization, one had trained as a doula, and others offered informal fertility-related support to those in need:

*If somebody said they were struggling, people would say ‘go and talk to [Marthe]’ because I’ve helped people with ovulation charts, temperature testing and all the stuff like that*.—Marthe

Finding new ways to support infertile couples made the end of their surrogacy journey easier for some participants:

*Working for the surrogacy organisation helped me stop being a surrogate because I felt I was still doing something just as good*.—Lillian

#### Not who people think we are

Although participants felt that people who become surrogates all share some personality traits, they were keen to communicate that surrogates come from ‘*all walks of life*’ (Lilian). This diversity was felt to be missing from public perceptions of surrogacy. More specifically, participants reported that the public wrongly assume that surrogates are women who have been exploited by those with greater socioeconomic power and education:

*There is a stigma around surrogacy that there is a certain class that is exploited by the higher classes for financial reasons. I think they feel that people are being exploited. But these are reasonable humans who understand the consequences of carrying a pregnancy*—Yvonne

*There’s a massive perception that surrogates are all poor, below the poverty line, uneducated, stupid, that they aren’t feminists, and they haven’t got a voice… that’s the thing that really pisses us off to be frank. It's really annoying that we apparently don’t know our own minds, and that we’re being coerced into it by rich gay men. It’s just patronising to be perfectly honest*.—Hazel

As alluded to by Hazel, participants disagreed with one school of feminist thought that viewed surrogacy as another way women’s bodies are utilized and commercialized by those with greater power at the expense of the bodily autonomy of the surrogate:

*There is still stigma from… people claiming that they are feminists telling us we shouldn’t be dictated to about our bodies. Well, we’re not. We’re making the decisions, not being forced.*—Alison

The misconception that surrogates are being exploited was understood to arise from the public being unaware that surrogates are not usually paid in the UK:

*The main question that I still get asked is ‘how much did you get paid to be a surrogate?’ I tell them that it’s actually illegal to get paid for surrogacy in the UK, then they say ‘but I saw this thing on the TV where a woman sold her baby for $40,000.’ They think you were coerced into it for money, and I say no… I just did this to help someone*.—Rose

Like Rose, Ruby described peoples’ disbelief that surrogates undergo the process for altruistic reasons: ‘*they can’t get their heads round the fact that people do it for no financial gain*’ *(Ruby*).

Many participants blamed public misconceptions about who surrogates are on inaccurate and negative media representations of surrogacy in the media. In TV and film, participants felt surrogates were largely represented as ‘*money grabbers*’ (Rhea) who had ‘*sold their baby*’ (Hannah), with surrogates feeling that these negative representations biased the public against surrogacy:

*For soap opera story lines… they would make it all about money and, ‘are they going to keep the baby’, and you think, why? That just makes it so much harder to make people understand what a positive thing [surrogacy] is.*—Alison

#### Made me who I am

Despite approximately two decades having passed since participants had experienced their first surrogate pregnancies, the majority of participants reported that undergoing surrogacy continued to be influential on who they are as a person. Often, this was because helping people by being their surrogate continued to have a positive impact on their feelings of self-worth:

*Sometimes I think my head’s going to get so big I won’t fit through the door. After all these years I still get them messaging me saying ‘I think of you every day, you’ve given me such a big gift’, and I think ‘oh my god, I’m so wonderful!’*—Annette

Participants reflected that their surrogacy journeys had also been a time for positive personal growth: ‘*it taught me a lot about me*’ (Adriana); ‘*it has changed me for the better*’ (Rose). Others went further to say that surrogacy had ‘*made me who I am*’ (Eloise). For Emilia, negotiating the unique challenges of being a surrogate had built up her own resilience, which helped her when she encountered a difficult situation later in her life:

*Because I’ve done something that is out of the ordinary, it makes other out of the ordinary things feel more possible… If I can do one thing that seems surprising, then why can’t I do another thing that seems surprising*.—Emilia

#### Part of my identity

Most participants described surrogacy as remaining at least somewhat important to their identity up to two decades after the surrogate pregnancy. Surrogacy had required a large investment of time and effort, particularly for those who underwent multiple surrogacy arrangements (as surrogacy had been an active part of their everyday lives for longer), meaning that surrogacy was seen as something that will ‘*always be with me*’ (Belinda) making it ‘*still a major part of my identity now*’ (Hazel). Participants did reflect that investing too much in their identity as a surrogate could pose challenges when they had to stop:

*I went through a period a few years ago of worrying that my identity and all my self-worth was wrapped around surrogacy. That was a bit of a concern. But I’ve learnt now that I’m still the same person… I still identify with it and it’s something I’m proud of… it’s not who we are, it’s what we were*—Clara

As time passed, several participants described how they did not brag about being a surrogate because they wish to avoid excessive external praise for something they do not perceive as particularly remarkable: ‘*I didn’t want to be put up on a pedestal or anything*’ (Zoe). Similarly, some participants described how their identity is complex and being a surrogate is one part of that identity. For these participants, they did not feel defined by surrogacy, but understood being a surrogate as one of the many things that made them who they are:

*It is part of my identity. When I think about myself, I think I’m a mum, I’m a daughter, I’m a girlfriend, I’m a this I’m a that, I’m a surrogate… it’s in the list of who I am and what I identify as.*—Rose

## Discussion

This analysis offers the first in-depth exploration of the long-term experiences of UK surrogates on average 20 years after their surrogate pregnancies for heterosexual couples. Though a minority of subsequent pregnancies were for single men or gay couples (N = 2). The findings reveal that those surrogates who were in contact with the families felt comfortable with the long-term relationships they had maintained, and all surrogates had incorporated their experiences as surrogates into a stable aspect of their identity. However, surrogates encountered physical, emotional, and interpersonal challenges long after the surrogacy birth(s). This ‘hard work’ was understood as integral to what it means to be a surrogate. The rewarding aspects of being a surrogate, namely the joy of seeing an infertile couple have a baby and gaining a network of friends through surrogacy, meant that managing the challenges of surrogacy was felt to be ‘worth it’. Surrogacy remained something participants were proud of, and most felt that being a surrogate still formed a positive part of their identity decades later.

Existing research exploring the experiences of egg and sperm donors shows that most UK identity-release donors feel their role requires them to stand back and allow the family autonomy, whilst remaining responsible for providing information to the child should they seek it (Graham *et al.*, 2016; Graham, 2022). Although typically maintaining closer relationships with the parents and children/young adults than is the case for gamete donors, similarities exist with how surrogates view their role in creating a new family. As seen elsewhere in the literature, surrogates did not view the child as ‘theirs’ (Yee *et al.*, 2020), and some enforced space between the family and themselves immediately following the birth to encourage the parents to feel ownership over their child. However, when the children grew up and had questions about their conception, some surrogates felt it was their responsibility to support the parents in these conversations. Surrogates stated that they could never imagine keeping a baby from the intended parents but were sympathetic towards the anxieties new intended parents felt. Surrogates hoped that by sharing their perspective, they would help future intended parents feel more able to trust their surrogate, and indeed these findings should prove reassuring to prospective intended parents considering surrogacy in the UK.

Becoming a surrogate was found to be something that first, affirmed surrogates’ existing identity as someone motivated to help others and second, positively impacted their identity over time. This is perhaps unsurprising, given the substantial body of psychological literature showing a positive association between prosociality (acting co-operatively and compassionately) and positive wellbeing (Hui *et al.*, 2020). As such, surrogates felt frustrated that negative stereotypes of surrogates as either ‘desperate victims’ or ‘money grabbers’ dominate UK media coverage of surrogacy (Gissen, 2023), misrepresenting their motivations for, and lived experience of, being a surrogate (van den Akker *et al.*, 2016). In reality, participants described that surrogates come from ‘all walks of life’ and are unified by a shared passion for helping people, having the resilience to handle the challenges involved in surrogacy, and possessing the firm belief that they can and will hand over the baby when the time comes.

The act of choosing to relinquish a baby challenges traditional ideals of womanhood and motherhood (Freeman *et al.*, 2014). There is a long history of debate between feminists who see surrogacy as inherently exploitative and those who think women should be able to make their own choices over their bodies (Teman, 2008). Some feminist writers oppose surrogacy because they feel it propagates repressive patriarchal and technological ideologies that pregnancy is merely a mechanical process to produce a baby, and surrogates are ‘used’ to reach this goal. They wish to see change in how western society views motherhood, promoting reconnection of womens’ bodies with their minds during pregnancy (Rothman, 1990). Interestingly, as theorized by Teman, some surrogates in this study reported feeling misunderstood by ‘so-called feminists’ who harboured assumptions about surrogates’ (in)capacity to make informed choices over their bodies. Perceiving stigma towards oneself is known to have negative implications for psychological health (Schmitt *et al.*, 2014). As such, surrogates wished to be understood in the media, research, and legislation as ‘reasonable humans’ who have made an active choice to become a surrogate. They hoped that such a shift would reduce the ignorance and discrimination they had experienced as surrogates in the past two decades.

Findings from the study show that feeling supported by the intended parents had a positive impact on surrogates’ experiences through the surrogacy journey. This finding corroborates existing research that investigated surrogates’ mental health during the COVID-19 restrictions and saw more positive outcomes when support was offered to surrogates from the intended parents (Yee *et al.*, 2023), and research on relationship quality and surrogate experiences in international surrogacy arrangements (Kneebone *et al.*, 2022). It is important for parents to recognize that surrogates have needs that will change with their own life circumstances, and the most positive outcomes are expected when all parties remain responsive and flexible in the relationship over time. When mutual support is offered, surrogates described how their relationship with the couple became like any other friendship. Having numerous, high-quality friendships is consistently found to predict positive wellbeing levels in non-surrogate populations (Pezirkianidis *et al.*, 2023), and satisfaction with friendship quality is significantly associated with life satisfaction independent of satisfaction with other relationships (Kaufman *et al.*, 2022). For many surrogates, the friendships they developed were felt to be one of the biggest rewards of their surrogacy experience. That friendship has been found to occupy a more central role in adults in midlife (46–65 years) than in established adulthood (30–45 years) (Schmidt *et al.*, 2022), emphasises the significance and intentional nature of these relationships to the surrogates who took part in the study. However, sustaining these open relationships required parents and surrogates to continue to work at their relationship and maintain flexibility in their expectations. The quality of the surrogate–parent relationship often changed over time, and this impacted how surrogates felt about their surrogacy arrangement, which often meant that surrogates who had helped multiple couples felt differently about each arrangement. Additionally, it is important that researchers and policymakers appreciate that the triadic relationship between parents, surrogate, and the child/young adult born through surrogacy will be ongoing and dynamic when developing support systems for surrogates and considering changes to legislation.

Our findings show that surrogates continue to feel the physical implications of their surrogate pregnancies long after the birth(s), suggesting that undergoing multiple surrogacy pregnancies may take a toll on surrogates’ physical health in the long term. Research on the impact of carrying multiple pregnancies on surrogates’ physical health is limited, but one clinical report suggests that the risk of gestational diabetes is higher in women who had carried more than three pregnancies (Liu *et al.*, 2020). In the UK, there are no limits currently to the number of times that surrogates can act as surrogates, and thus it may fall on surrogacy agencies and clinics to advise surrogates about any potential risks. Findings from the present study suggest that healthcare professionals may also wish to inform potential surrogates about the emotional and physical toll that surrogacy may take, and that the interpersonal and emotional work continues for some surrogates long after the surrogacy birth. Further research is required to identify what specific support should be offered at different time points on their surrogacy journey to best support surrogates in the years following a surrogacy.

The in-depth, qualitative approach taken in this study offers novel insight into the experiences of surrogates in the decades following a surrogacy. To the best of our knowledge, it is the only study to examine surrogates’ experiences more than 20 years since their first surrogacy arrangement(s). The sample size can be considered relatively large for an analysis of this depth, and the large number of surrogacy arrangements experienced and discussed by participants (n = 71) is a further strength of the research as it allows for variety of experiences. There is a risk of selection bias, with surrogates who had a positive experience being more likely to continue to participate in the research. However, the participation rate for the current study was high and the diversity observed in the narratives suggests that the sample reflects both the challenges and benefits of being a surrogate. It is also worth noting that in the UK, where this research was undertaken, there is no limit on the number of times that a woman can carry a surrogate pregnancy, whereas other jurisdictions may place limits on this as may some clinics. As a result of this, some of the surrogates’ reflections on surrogacy, particularly on the physical toll of multiple surrogacy pregnancies, may be specific to this context. Most of the surrogacies were carried for heterosexual couples and it is possible that surrogates’ relationships may differ with gay couples and single men. While data are available on the quality of relationship between surrogates and gay couples from all parties’ perspectives in the short-term (Blake *et al.*, 2016; Jadva *et al.*, 2019), nothing is known about relationships over the longer term. Although the study collected data on whether surrogates had engaged in traditional or gestational surrogacy, data on whether a donor egg or the intending mothers’ egg was used for gestational arrangements were not collected.

In conclusion, surrogates continued to reflect positively on their experiences of being a surrogate decades after they gave birth. There are many physical, emotional, and interpersonal challenges involved in being a surrogate, and these can continue long after the surrogacy arrangement. Traditional ideas of motherhood remain pervasive, meaning surrogates and intended parents may expect to feel their route to parenthood challenges social norms. Prospective parents and surrogates also ought to expect significant work to be involved in building and maintaining positive relationships with each other throughout their surrogacy journey. However, enjoying these relationships and feeling proud of the barriers overcome were experienced as rewarding. By the time children born through surrogacy reached young adulthood, most surrogates continued to feel that surrogacy formed an important, although not primary, part of their identity.

## Data Availability

The data underlying this article cannot be shared publicly in order to maintain the privacy of individuals that participated in the study.
